# Bottom of the Heap: Having Heavier Competitors Accelerates Early-Life Telomere Loss in the European Starling, *Sturnus vulgaris*

**DOI:** 10.1371/journal.pone.0083617

**Published:** 2013-12-27

**Authors:** Daniel Nettle, Pat Monaghan, Winnie Boner, Robert Gillespie, Melissa Bateson

**Affiliations:** 1 Centre for Behaviour and Evolution & Institute of Neuroscience, Newcastle University, Newcastle, United Kingdom; 2 Institute of Biodiversity, Animal Health & Comparative Medicine, University of Glasgow, United Kingdom; Utrecht University, Netherlands

## Abstract

Early-life adversity is associated with poorer health and survival in adulthood in humans and other animals. One pathway by which early-life environmental stressors could affect the adult phenotype is via effects on telomere dynamics. Several studies have shown that early-life adversity is associated with relatively short telomeres, but these are often cross-sectional and usually correlational in design. Here, we present a novel experimental system for studying the relationship between early-life adversity and telomere dynamics using a wild bird, the European starling (*Sturnus vulgaris*). We used cross-fostering to experimentally assign sibling chicks to either small or large broods for twelve days of the growth period. We measured telomere length in red blood cells using quantitative PCR near the beginning of the experimental manipulation (4 days old), at the end of the experimental manipulation (15 days old), and once the birds were independent (55 days old). Being in a larger brood slowed growth and retarded wing development and the timing of fledging. We found no evidence that overall brood size affected telomere dynamics. However, the greater the number of competitors above the focal bird in the within-brood size hierarchy, the greater was the telomere loss during the period of the experimental manipulation. The number of competitors below the focal in the hierarchy had no effect. The effect of heavier competitors was still evident when we controlled for the weight of the focal bird at the end of the manipulation, suggesting it was not due to retarded growth per se. Moreover, the impact of early competition on telomeres was still evident at independence, suggesting persistence beyond early life. Our study provides experimental support for the hypothesis that social stress, in this case induced by the presence of a greater number of dominant competitors, accelerates the rate of telomere loss.

## Introduction

Adverse environmental conditions experienced in early life can be associated with increased mortality and morbidity once the individual is an adult. For example, in humans, the risks of a wide range of health problems, as well as early death, are higher in people who, many years earlier, experienced childhood socioeconomic deprivation [1], [2], parental divorce [3], large sibship size [4], or parental abuse and neglect [5], [6]. Researchers have become increasingly interested in the biological pathways that mediate these long-term impacts of developmental history [7], [8]. A potential route for environmental adversity to have long lasting effects on the developing individual is via changes in telomere dynamics. Telomeres are non-coding, repetitive DNA sequences at the ends of the linear chromosomes of eukaryotes. Telomeres identify the chromosome ends, prevent end-to-end joining, and also protect the coding sequences from the loss that occurs at the chromosome ends during DNA replication. In many vertebrate somatic cells, telomeres shorten with each round of cell division, and thereby get shorter as the individual ages, with the fastest rate of shortening occurring early in life [9]–[11]. When telomeres reach a critically short length, cells enter a state of replicative senescence, following which they either die or show a changed secretory profile with increased secretion of inflammatory compounds [12]. Short telomere length in a cell or tissue population is therefore associated with increased likelihood of malfunction. Thus, increased telomere attrition potentially underpins the lasting effects of the early environment on the phenotype that can lead to poorer health outcomes later in life [13], [14].

In humans, leucocyte telomere length has been found to prospectively predict survival and health [15]–[18]. In birds, telomere length, usually measured in red blood cells, which are nucleated, is a strong predictor of subsequent survival, with telomere length at the end of the growth period having the strongest predictive power [11], [19]–[21]. Telomere loss is sensitive to the environment. The rate of telomere shortening is accelerated by oxidative stress [22], and may also be increased by exposure to stress hormones [23]. In humans, current and cumulative life stress [24], [25] or a history of childhood adversity [26]–[28], have been found to be associated with relatively short telomeres.

Human studies on the effects of early environment on telomeres necessarily suffer from being correlational, and are usually genetically uncontrolled, which is important since there are likely to be large genetic influences on TL [29], as well as on mortality and morbidity. Birds, by contrast, allow for the possibility of experimental manipulation of early experience. Altricial birds are good models for studying the links between environmental conditions, telomere dynamics and aging because of their developmental immaturity at hatching, considerable longevity, and somatic down-regulation of the telomere restoration enzyme telomerase [30], features that they share with humans. For example, maximum recorded longevities for European starlings in the wild are 15 years (North America) and 21 years (Germany), several times what would be seen in a mammal of similar mass [31].

Salomons, Mulder and Verhulst [32] experimentally enlarged or reduced the broods of jackdaws *Corvus monedula* and examined the effect on average telomere length within broods. In male chicks only, being in an enlarged brood led to significantly accelerated telomere loss over the first 25 days post-hatching. This effect was not reducible to the slower weight gain of chicks in the enlarged broods, suggesting that it reflects different exposure to social stress in broods of different sizes. Nestling competition in birds involves begging, jostling for position and inter-nestling aggression, all of which are increased in larger broods. Increasing brood size is associated with increased intra-nest variance in chick sizes [33]. This arises both because parents preferentially feed larger chicks, and also because larger chicks are better able to compete against their siblings for prime nest positions [34]–[36]. In the resulting size hierarchy, the smaller chicks have to work harder than their siblings to obtain food [34], and experience higher levels of physiological stress [37]. Even though offspring weights often converge by the end of the nestling period, there can be lasting impacts on the fitness of individuals who were lower in the size hierarchy [38]. Thus, if the degree of social stress is an important factor, accelerated telomere loss might not affect all chicks growing up in large broods, but should be most evident in those in individuals in lower positions in the size hierarchy.

In this study, we investigated effects of nestling competition and position in the size hierarchy on early-life telomere dynamics in wild European starlings *Sturnus vulgaris.* European starlings are colonial, cavity-nesting passerine birds widely used in biological research [39], [40]. We used a full cross-fostering design in which quartets of siblings were removed from their natal nests two days after hatching. Two siblings were moved to a foster nest that contained five other competitor chicks, thus creating a highly competitive environment; the other two were moved to a nest where they were the only chicks, and thus competition was low. The chicks remained in their experimental broods for 12 days before being taken into captivity. By using siblings, our design controlled for genetic and *in ovo* effects. We tracked the weights not just of our focal individuals, but also of the other chicks in the large broods. This allowed us to ascertain the position of the focal chicks within the size hierarchy of the nest. We measured relative telomere length by quantitative PCR (qPCR) near the beginning of the experimental manipulation, at the end of the manipulation period, and after they had fledged and reached independence. Chicks were also genetically sexed in order to test for interactions between sex and competition.

## Methods

### Ethics statement

Our study adhered to the Association for the Study of Animal Behaviour (ASAB) Guidelines for the Use of Animals in Research, and was approved by the local ethical review committee at Newcastle University. It was completed under UK Home Office project licence number PPL 60/4073 (Melissa Bateson), and removal of starlings from the wild was authorised by Natural England (licence number 20121066). Invasiveness of field research was minimized as described below, and husbandry for starlings in captivity complied with advice in the Universities' Foundation for Animal Welfare (UFAW) *Care and Management of Laboratory and Other Research Animals* handbook [31]. All fieldwork on farms was carried out with the permission and kind assistance of the farmers.

### Study species and brood size manipulation

We studied wild European starlings nesting in colonies on five farms in Northumberland, Northeast England, in the breeding season of 2012. Accessible starling nesting boxes have been installed at these sites for a number of years. We monitored egg-laying daily in order to identify sets of nests in which chicks were likely to start hatching on the same day. Within a nest, starling chicks hatch on the same day with the exception of one late-laid egg that hatches a day later; none of these late hatches were used as focal birds, thereby minimising as much as possible within-brood variation in the quality of the hatchlings involved in the experiment. All nests involved in this study started hatching within four days of one another, thus minimising between-brood variation in parental quality or age, which is generally reflected in laying date [41].

Using a digital balance, we weighed all chicks on the day after hatching, and selected donor nests containing at least four chicks of approximately the same weight which became our focal chicks. On post-hatching day three (D3, where D1 is day of hatching), we moved two of each set of focal chicks to a host nest where they would be the only nestlings, whilst the other two were moved to a different host nest where we also placed five additional competitors. Nests of seven chicks are within the observed range of natural variation in this population of starlings, and this manipulation has been used previously without causing chick mortality [33]. The additional competitors in the large brood nests were not siblings of the focals, and also were not in their natal nests. Thus, no host parent in the study raised any of their own chicks. Assignment to small or large brood conditions was random. Chicks were out of the nest for the minimum possible time and kept warm during transport. To minimize risk of parental desertion, nest boxes were never left empty of chicks. Surplus chicks remaining after the cross-fostering operations were put into donor nests to replace outgoing focals. Thus, all chicks that hatched were housed in a nest, and all parents received a brood of chicks.

We created nine sets of four focal siblings (36 birds) in this way, but one small brood was abandoned on D4 and the chicks died. In large broods, where a non-focal competitor died within the first three days post-manipulation, we replaced the dead individual with another chick of approximately the same weight. Due to mortality at later stages, one of the large broods contained only 6 live chicks on D15, and one nest 5. We weighed the focal chicks on D4, D7, D11 and D15. In addition, on D15, we weighed all of the non-focal competitor chicks from the large broods. We measured tarsus lengths and wing lengths for the focal chicks on D15 using digital calipers and a wing rule respectively. Each tarsus was measured twice independently. Correlations between the two measurements on the same side were 0.99 (right) and 0.97 (left), with the mean difference between the first and second measurement 0.16 mm (s.d. 0.28) for the right and 0.03 mm (s.d. 0.30) for the left. The correlation between the mean of the left and the mean of the right measurements was 0.94. Tarsus lengths reported here represent the mean of the four measurements. Left and right wing lengths were correlated at *r* = 0.98, and the measurements reported here represent the mean of the two sides.

### Hand-rearing

On D15, the surviving focal chicks were taken from their nests and reared in captivity for subsequent behavioural study. In large broods, non-focal chicks were left in the nest, and in small broods, parents were given two chicks of the correct size from other nests nearby to rear to fledging; no nests were deserted as a result of this second brood manipulation. Once in captivity, the four individuals from each natal family were put back together in a covered bucket containing a tissue-paper nest, were they were hand-reared until fledging at around D21. Birds in buckets were fed to satiation on commercial poultry-based cat foods mixed with apple sauce and added vitamins and minerals (full details of hand-rearing methods are provided in [42]). Unfortunately, due to their age, the birds did not imprint on humans and had to be force fed for the duration of hand-rearing. After fledging (defined as bird flying from bucket), birds were group-housed, initially in cages and later (around D30 onwards) in large free-flight rooms. The fledged birds were fed *ad libitum* on commercial poultry-based cat foods, fruit, commercial grain-based chick starter crumbs, live mealworms (*Tenebrio molitor*) and dried insect pate (Orlux). Birds were weighed in captivity at D20 and D55 (+/− 2 days). Their day of fledging from the buckets was also recorded. One bird died after fledging but before D55.

### Blood sampling and telomere analysis

We used the T/S ratio from a quantitative PCR analysis as our assay of telomere length [43]. Due to its low cost and DNA requirement per sample, this technique has rapidly become a standard methodology for epidemiological and ecological studies of telomere dynamics [e.g. 21,24,25–28]. Results from quantitative PCR are very highly correlated with those produced by Telomere Restriction Fragment Analysis [44]. The T/S ratio is a an average measure across all cells in the sample, and is a relative measure of telomeric sequence abundance within the genome rather than an absolute measure of length in base pairs. Thus, the T/S ratio includes the abundance of interstitial telomeric sequences as well as those located at chromosome ends [45]. Some bird species have relatively high numbers of interstitial repeats of the telomeric sequence, and variation in these makes it more difficult to see cross-sectional effects with the quantitative PCR method [46]. This issue is however relatively inconsequential for the current study where we had repeat measurements from the same individuals over time, since the abundance of interstitial telomere sequences should be stable within an individual. Thus, changes over time are likely to be largely due to telomere attrition at chromosome ends.

We extracted a maximum of 75 μl of blood from the alar vein of each focal on D4 (one day into the experimental manipulation), D15 (the end of the experimental manipulation) and D55 (approximately 25 days after independence and living in free-flight aviary), using a sterile needle and heparinized capillary tube. We applied antiseptic cream to the puncture site, and no birds suffered detectable adverse consequences as a result of blood sampling. Samples were placed on ice, and within three hours centrifuged to separate cells from plasma. Cells were then frozen to −80°C until DNA extraction and qPCR analysis of telomere length. The blood samples taken at the different time points were all analysed at the same time.

Genomic DNA was extracted from red blood cells using the MACHEREY-NAGEL Nucleospin® Blood Kit (MACHEREY-NAGEL GmbH & Co. KG, Düren, Germany) by resuspending 3–4 μl of red blood cells in 196 μl of PBS and following the manufacturer's protocol for DNA purification from whole blood. The concentration and quality of DNA samples were assessed using a Nanodrop-8000 Spectrophotometer; only samples with A260/280>1.8 and an A260/230>1.9 were assayed. DNA samples were stored at −20°C.Relative telomere measurements were made using the qPCR methods as described by Criscuolo et al. [47] with the following modifications. DNA samples (10 ng) were assayed using the Absolute blue qPCR SYBR green Low Rox master mix (Thermo scientific) with telomere primers (Tel1b and Tel2b) at a final concentration of 500 nM and Gapdh primers (GapF and GapR) at a final concentration of 70 nM. The telomere thermal profile was 15 minutes at 95°C, followed by 27 cycles of 15 seconds at 95°C, 30 seconds at 58°C, 30 seconds at 72°C. The Gapdh thermal profile was 15 minutes at 95°C, followed by 40 cycles of 15 seconds at 95°C, 30 seconds at 60°C, 30 seconds, 72°C. Both assays were followed by melt curve analysis of (58–95°C 1°c/5 s ramp).The reference sample was serially diluted (from 40 to 2.5 ng/well) to produce a standard curve for each plate. This was used to calculate plate efficiencies, all of which fell within the acceptable range (i.e. 100±15%) and only samples that fell within the bounds of the standard curve were included. Each sample was assayed in triplicate and the mean of the three assays used. All samples from the same individuals were assayed on the same plate, but nests and treatments were randomized across different plates.

Nine individuals repeatedly fell out with the Gapdh standard curve and were therefore excluded from the analysis. Gapdh is the single copy gene that is used to normalise the input and as 10 ng of DNA is loaded per well, we would expect sample amplification to fall around the 10 ng point of the standard curve, as was the case for all the other samples in the study. The telomere amplification of all samples from these individuals did not fall within the standard curve suggesting something unusual about the chromosomes of these individuals, four of which were from the same natal nest.

As described in [47], relative telomere measurements were calculated using the ΔΔCt method. This provides a ratio of the abundance of the telomeric sequence to the abundance of the reference single copy gene (henceforth, T/S ratio). There were no overall differences in T/S ratios between the samples run on different plates (F_4,19_ = 0.86,p = 0.51). The average intraplate variation of the Ct value was 1.23% for the telomere assays and 0.22% for the Gapdh assays, and the average interpolate variation of the ΔCt was 1.2%.

### Sex determination

Molecular sexing was carried out by amplification of the chromodomain-helicase-DNA binding (CHD) genes in 10 μl PCR reactions. Final concentrations of reagents were 1X Green GoTaq® Flexi Buffer (Promega), 2 mM Magnesium chloride (Promega), 0.8 mM dNTPs (Promega), 0.8 uM 2550F (5′-GTTACTGATTCGTCTACGAGA-3′) [48], 0.8 uM 2757R (5′-AATTCCCCTTTTATTGATCCATC-3′) (Griffiths, unpublished data), 0.375 U GoTaq® DNA Polymerase, and approximately 100 ng of DNA. Volumes were brought to 10 μl with H_2_O.The thermal cycle profile for the PCR comprised 94°C for 2 minutes, followed by 30 cycles of 49°C for 1 minute, 72°C for 1 minute, 94°C for 45 seconds, with a final cycle of 49°C for 2 minutes and 72°C for 5 minutes. PCR products were separated on a 2% agarose gel, with two bands indicating the presence of a Z and W chromosome (female), and one band indicating the presence of only the Z chromosomes (male).

### Statistical analysis

**Figure 1:**
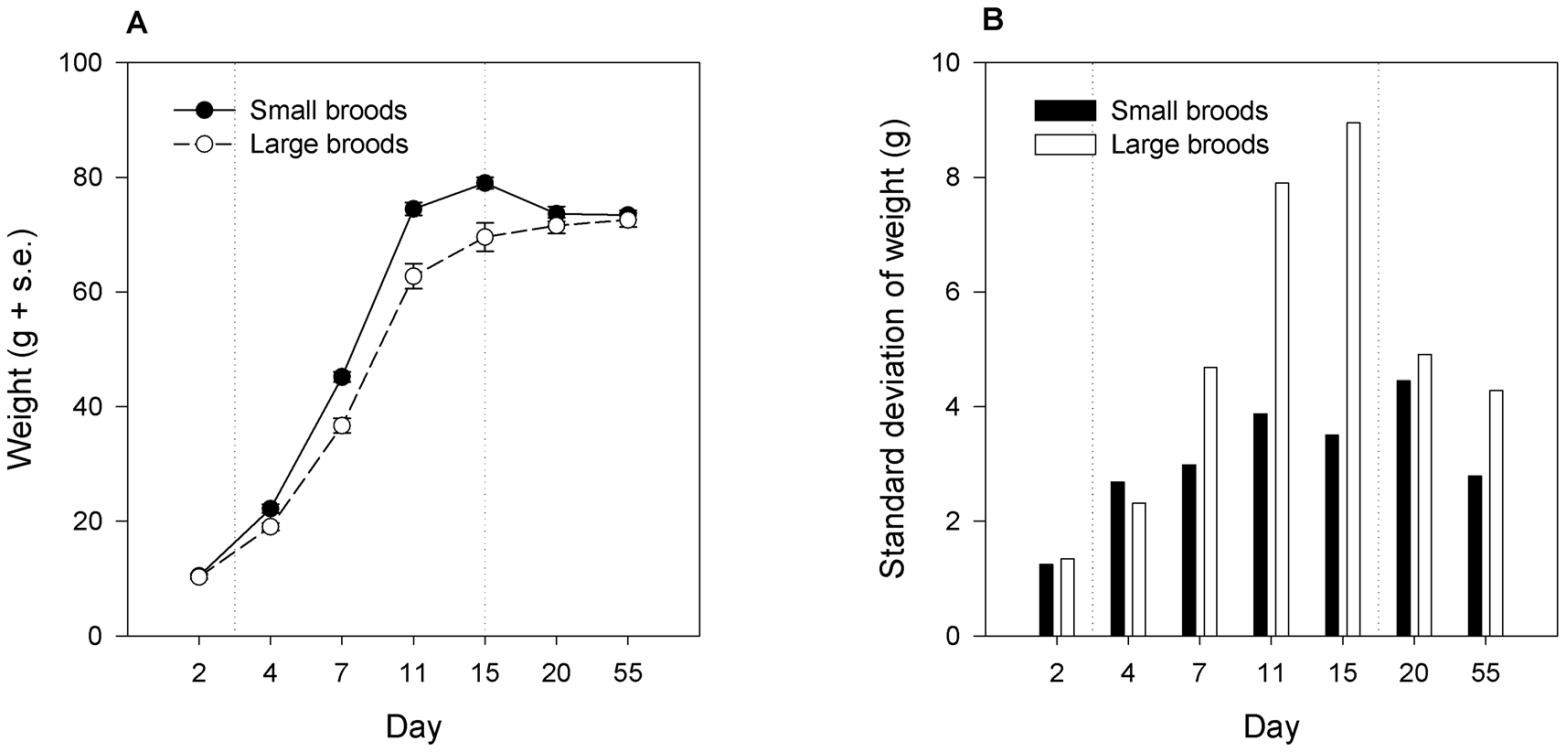
Effects of nestling competition on growth. The vertical lines represent the beginning and end of the period in the experimental broods. A. Means and standard errors for weights over time for birds assigned to large broods (5–7 chicks) and small broods (2 chicks). B. The standard deviations at each time point of the weights of birds assigned to large and small broods.

**Figure 2:**
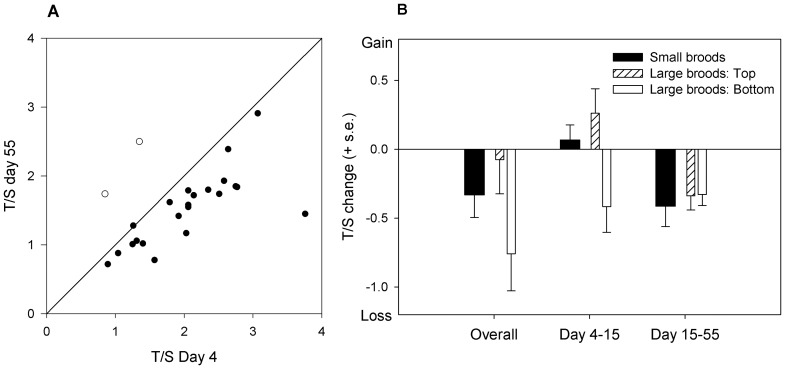
Telomere dynamics in the starlings. A. T/S ratio at day 55 plotted against T/S ratio at D4. Each point represents a bird, and the solid line is y = x. The two birds represented by unfilled circles are the only ones to have lower T/S ratio at day 4 than at both subsequent time points, and are those discussed in the text as outliers. B. Mean T/S ratio change for the study period overall (day 4 to day 55), for the period of the experimental manipulation in the nests (day 4 to day 15), and for the post-manipulation period in captivity (day 15 to 55). Birds from the large broods are sub-divided into those who occupied the first or second place in the within-brood size hierarchy (‘top’) and those who occupied lower positions (‘bottom’).

Data were analysed using linear mixed models in SPSS version 19.0 using full maximum likelihood estimation. Only data from birds with T/S values for D4 and at least one of D15 and D55 are included in analyses presented here (n = 25). All models reported below included a random effect of natal family. The main repeated measures models reported below used an autoregressive (AR1) covariance matrix suitable for data representing change within an individual over time. Outcomes were unchanged if a diagonal covariance matrix was used instead. Because not all of our large brood nests contained 7 live chicks at D15, for statistical analysis we used actual brood size at D15 as the continuous independent variable. Using large vs. small brood as a dichotomous variable produced essentially identical results. For ease of reading we used the large-versus-small brood dichotomy to produce figures 1 and 2b and for giving illustrative means, but the statistical analysis always used the continuous variable. To examine the effects of position within the size hierarchy, we subsequently divided number of competitors at D15 into the number of competitors heavier than the focal, and the number lighter than the focal, based on weights at D15.

## Results

Data from the study are downloadable as File S1.

### Effects of experimental manipulation on growth

Weights were available from D2 (pre-experimental manipulation), D4, 7, 11 and 15 (during experimental manipulation), D20 and D55 (post-experimental manipulation). In a repeated measures model with weight as the dependent variable, time point, brood size and their interaction as fixed effects, and natal family as a random effect, there was a significant main effect of time point (F_6,86.21_ = 230.56, p<0.01), a significant main effect of brood size (F_1,24.63_ = 15.60, p<0.01), and a significant time point by brood size interaction (F_6,86.21_ = 8.18, p<0.01). This analysis spans two distinct phases of weight gain, the experimental period in the nest, and the period of hand-rearing in the laboratory, and the significant interaction with time point might be caused by the equalizing of conditions after D15. We therefore repeated the model using just weights from D2, 4, 7, 11 and 15. Again, there was a significant main effect of time point (F_4,91.98_ = 238.19, p<0.01), a significant main effect of brood size (F_1,29.43_ = 19.94, p<0.01), and a significant time point by brood size interaction (F_4,91.98_ = 5.89, p<0.01). As figure 1a shows, birds in large broods were no lighter than birds in small broods prior to the experimental manipulation, but grew significantly more slowly through the manipulation period, and then converged in weight after D15.

As well as slowing average growth, larger brood size also increased heterogeneity between birds. Figure 1b shows the standard deviation in weights over time for birds in large and small broods. Birds in large broods had higher standard deviations during the manipulation period, especially at D11 and D15, though on none of the individual days was the difference in variability significant on a Levene's test (data not shown). Five of the focal birds in large broods were in the top one or two positions of the within-brood size hierarchy at D15, and their weights were only marginally lower than those of the birds in the small broods. It was the eight birds who were both in large broods and lower-placed in the within-brood size hierarchy whose growth was substantially different from their siblings in the small broods (mean ± s.e. weights D15, birds in small broods 78.98±1.01 g; top birds in large broods: 76.52±2.34 g; bottom birds in large broods 65.23±2.86 g).

Our sample contained 11 male and 14 female birds. When sex was added to the repeated measures model predicting weight over time, there were no significant effects of sex, either as a main effect or in interaction with time and/or number of competitors (data not shown). Thus, there was no evidence that males and females grew differently or responded differently to competition.

Tarsus length at D15 was shorter in birds from larger broods, but not significantly so (model with brood size as a fixed effect and natal family as a random effect: F_1,18.75_ = 2.27, p = 0.15, mean ± s.e. for small vs. large broods 34.38±0.22 mm vs. 33.48±0.49 mm). Birds from larger broods had significantly shorter wings at D15 (model as previous: F_1,19.01_ = 22.36, p<0.01, means ± s.e. for small vs. large broods 80.60±0.68 mm vs. 74.23±1.32 mm). As for weight, there was heterogeneity within the large broods, with the reduced wing length particularly marked in the birds that were in lower positions in the within-brood size hierarchy (top birds in large broods: 76.60±1.03 mm; bottom birds in large broods 72.75±1.91 mm). Birds from larger broods also fledged significantly later than those from smaller broods (model as previous: F_1,17.55_ = 14.08, p<0.01, means ± s.e. for small vs. large broods 19.83±0.37 days vs. 21.31±0.37 days). This delay was essentially restricted to the eight individuals who were in lower places in the within-brood size hierarchy at D15 (top birds in large broods: 20.40±0.37 days; bottom birds in large broods 21.88±0.40 days).

### Telomere dynamics

T/S ratio measurements from the same individuals were correlated over time (D4 to D15, r = 0.72, p<0.01; D15 to D55, r = 0.73, p<0.01; D4 to D55, r = 0.54, p<0.01). Across the sample, the mean T/S ratios from quantitative PCR were 1.99 (s.d. 0.73) at D4, 1.90 (s.d. 0.50) at D15, and 1.57 (s.d. 0.54) at D55. Figure 2b plots T/S ratio at D55 against T/S ratio at D4 for each individual. All but two of the points lie on or below the y = x line, showing that individuals did generally have lower T/S ratios at the oldest time point, thus illustrating telomere shortening with age. The two individuals indicated with unfilled circles on figure 2a were outliers in that their T/S ratios were substantially lower at D4 than either of the later time points. Since these birds (one from each treatment) may represent measurement error, all subsequent analyses are repeated both for the full dataset and with these two birds excluded.

We first conducted a repeated measures analysis of T/S ratio at the three time points, entering time point, brood size and their interaction as fixed effects, and natal family as a random effect. There was a significant effect of time point (F_2,46.53_ = 3.29, p = 0.046), but no effect of brood size (F_1,24.86_ = 0.32, p = 0.57), nor time point by brood size interaction (F_2,46.49_ = 1.27, p = 0.29). Excluding the two outlier birds did not change this pattern of results (time point, F_2,28.80_ = 5.39, p = 0.01; brood size, F_1,22.99_ = 0.18, p = 0.67; time point by brood size interaction, F_2,28.48_ = 1.64, p = 0.21). To examine the effect of position in the weight hierarchy, we decomposed brood size at D15 into the number of competitors that were heavier than the focal, and the number that were lighter, and entered these terms and their interactions with time into the repeated measures model predicting T/S ratio. There was a significant main effect of time point (F_2,47.05_ = 5.57, p<0.01), and a significant time point by heavier competitors interaction (F_2,47.11_ = 6.72, p<0.01). All other main effects and interactions were non-significant (main effect of number of heavier competitors, F_1,25.21_ = 0.00, p>0.99; main effect of number of lighter competitors, F_1,25.18_ = 0.92, p = 0.35; time point by number of lighter competitors interaction, F_2,47.56_ = 0.65, p = 0.53). The significant effects persisted unchanged when the two outlier birds were excluded (time point, F_2,28.97_ = 8.64, p<0.01; time point by heavier competitors interaction, F_2,28.83_ = 5.67, p<0.01; all other effects p>0.10).

To visualize why number of heavier competitors significantly affected telomere dynamics whilst overall brood size did not, we plotted the mean decrease in T/S ratio over the study period for birds from the small and large broods, with the large brood-size birds sub-divided into those who were in the top one or two positions of the within-brood weight hierarchy (‘top’ birds) and those who occupied a lower position (‘bottom’ birds; figure 2b). Large-brood ‘top’ birds, who had many competitors lighter than themselves but few competitors heavier than themselves, had a smaller decrease in T/S ratio over the study period than small-brood birds. It was the large-brood ‘bottom’ birds, who had many competitors heavier than themselves, who had a markedly greater T/S ratio reduction compared to small-brood birds (figure 2b). Dividing the study period into the D4 to D15 (in the nests) and D15 to D55 (in captivity) sections, the difference in telomere attrition between the large-brood ‘bottom’ birds and the other groups was restricted to the period between D4 and D15 (figure 2b). Between D15 and D55, there was no further difference in telomere loss between the groups, though the increased telomere loss in the large-brood ‘bottom’ birds seen between D4 and D15 was not reversed.

When we added weight at D15 and its interaction with time point to the statistical model, the effects of weight D15 were not significant (main effect, F_1,25.06_ = 1.55, p = 0.22; interaction of weight D15 with time point, F_2,46.90_ = 0.45, p = 0.64), whilst the significant interaction between time point and number of heavier competitors persisted (F_2,46.91_ = 3.86, p = 0.03). This pattern was not changed with the two outlier birds excluded (weight D15, F_1,22.91_ = 1.43, p = 0.24; time point by weight D15 interaction, F_2,28.76_ = 1.10, p = 0.35; time point by heavier competitors interaction, F_2,28.88_ = 4.58, p = 0.02). We also ran models including pre-manipulation (D2) weight, and weight gain from D2 to D15, as covariates. Again, with or without the outlier birds, there were no significant effects involving D2 weight or weight gain, but the heavier competitors by time point interaction remained significant (data not shown).

### Sex differences in telomere dynamics and other covariates

Using as a base a repeated-measures model predicting T/S ratio with fixed effects of time point, number of heavier competitors and their interaction, and a random effect of natal family, we sequentially added genetic sex and each of its interactions with the other two variables. In no model was any effect involving sex significant, and in no model did the time by heavier competitors interaction become non-significant (data not shown). We similarly explored adding the biological parents' and host parents' original brood sizes, as indices of quality of the biological and host parents respectively, but in no model were these variables or any of their interactions significant, or the interaction between time and number of heavier competitors altered (data not shown).

## Discussion

We experimentally assigned starling siblings to spend 12 days of early life in nests either with just one competitor, or with many (4–6) competitors, and examined the consequences of this treatment for telomere dynamics over the first 55 days of life. Being in a larger brood dramatically affected growth, slowing mean weight gain, increasing within-brood variability in weight, and retarding wing growth and date of fledging. We found no evidence that brood size *per se* affected telomere dynamics. However, we did find evidence that the number of competitors heavier than the focal was important: the greater number of competitors heavier than the focal within the brood, the more telomere length that focal lost by D55 of life. This pattern was extremely robust to the exclusion of outlier birds and the inclusion of additional covariates into the analysis. The number of lighter competitors had no effect on telomere dynamics in any of our analyses. Thus, it appears that what affects telomere loss is not having competitors; it is having competitors relative to whom one is at a disadvantage. To reinforce this point, the birds that were in large broods but near to the top of the within-brood hierarchy showed no evidence of any telomere attrition at all during the period of the experimental manipulation.

Birds with a greater number of heavier competitors did not have shorter telomeres at D4, and their increased telomere loss was restricted to the period of the actual manipulation (D4–D15). It did not continue in the post-manipulation period (D15–D55) when all the birds were being hand-reared. Thus, it seems plausible that experiencing competition in which the focal was disadvantaged had a direct, immediate causal impact on telomeres, rather than the disadvantaged birds simply being different in some other way from the advantaged ones (for example, genetic quality). Although the accelerated telomere loss of the birds with a greater number of heavier competitors did not continue into the D15 to D55 period, neither was its effect reversed during this period. Thus, at D55, the impact of the 12 days of competition in the nest could still be clearly seen, even though those 12 days had been followed by 40 days of subsequent experience, experience that was uniform across all individuals, and presumably quite stressful, since the birds were in captivity.

The effect of heavier competitors on telomere dynamics was not reducible to poorer absolute weight gain, since it persisted once weight at D15 was controlled for. Number of heavier competitors was not perfectly correlated with weight at D15 because there was heterogeneity in weight gain between broods, so that a bird that was low in the weight hierarchy of its particular brood might in absolute terms be heavier than all the individuals from a different brood. The fact that it is the number of heavier competitors rather than an individual's weight that predicts telomere dynamics implies that the pathway linking early conditions to telomere dynamics is not via absolute growth parameters, but, rather, the consequences of relative position within the brood. In altricial birds, it is well documented that relatively smaller chicks within a brood have to struggle harder to obtain parental investment [34]–[36]. The competition that these subordinate chicks face may mobilize physiological stress mechanisms, whose effects are adaptive in the short-term, but costly in the long term, as they reallocate energy towards the immediate challenges of staying alive, and away from somatic self-repair [49]. Consistent with this view, physiological stress is associated with reduced antioxidant production and increased cellular oxidative stress [50], [51], which in turn damages telomeres [22], [52], and antioxidant capacity is reduced in birds growing up in large broods [53]. Thus, there is a plausibly physiological pathway, involving physiological and oxidative stress, by which being disadvantaged in the within-nest weight hierarchy could lead to the pattern of telomere dynamics we observed.

Our results are partly consistent with those of Salomons et al. [32] in jackdaws. They found that being in an experimentally enlarged brood increased telomere attrition over the first 25 days of life, for male chicks only. Our findings also show that manipulating nestling competition has effects on telomere dynamics, and like Salomons et al., we found that the effects were not due to absolute weight gain. Unlike Salomons et al., though, we found that only the number of heavier competitors, and not the number of competitors overall, mattered. We also did not find any evidence that the effect in starlings was sex-specific. However, although there is some sexual dimorphism in size in adult European starlings [31], sex differences in early development in starlings are minimal. In the current sample, we did not find any significant effects of sex on weight, either as a main effect, or in interaction with time or number of competitors. By contrast, Salomons et al. [53] found that in their population of jackdaws, there were significant sex differences in juvenile weight, growth trajectories, and in the response to increased brood size. Sex differences in response to early conditions are widely found in birds, but highly variable across species [54], [55], and starlings may differ from jackdaws in this regard.

The principal strengths of our study are that it was longitudinal, genetically controlled, and we had at least partial experimental control of early-life conditions. Studies of early-life adversity and TL in humans have mostly been cross-sectional in design [27], [28], making inferences about causality problematic. Longitudinal studies with telomere measurement before and after exposure represent an improvement in this regard [26]. Genetic variance in starting telomere length is also a source of error variation [29]. This was minimized in our study by the use of quartets from the same natal nest. While intra-specific brood parasitism is well-known in the European starling [56], [57], it tends to affect a minority of nests (15% on average in one long-term study [56]), and involve a single introduced egg. None of the donor nests in our study had two new eggs appear on the same day, a sufficient but not necessary sign of parasitism. Thus, we suggest that the vast majority of our sibling groups are likely to have been genetic siblings, although we did not test this assumption using genetic methods. The principal limitation of the study was our small sample size. Our sampling was constrained by using a wild-breeding animal and relying on finding trios of nests that hatched nearby at the same time. Our statistical power for detecting subtle interactions – for example, with sex – was modest.

We describe our experimental control of early-life conditions as partial rather than complete. We randomly assigned siblings to either small or large broods. We did not, however, have experimental control over where they ended up within the brood hierarchy of their experimental nest, and it turned out to be this rather than the brood size variable that we manipulated that was associated with telomere dynamics. In the large broods, position within the weight hierarchy at the end of the manipulation was partly attributable to initial differences in weight amongst chicks at the start of the manipulation (correlation between weight at D2 and number of heavier competitors at D15, r = 0.60, p = 0.03), though weight at D2 did not itself predict telomere dynamics. Since starlings weigh only around 5 g on hatching, and gain at least 6 g per day, the variation in weight at D2 might reflect the timing of hatching relative to our arrival to weigh the chicks as much as any intrinsic attributes of the chicks. Thus, it is possible that position attained within the weight hierarchy is mainly due to chance aspects of how we composed the experimental broods, in which case, it is effectively a randomly-assigned variable. However, we cannot fully exclude the possibility that there might have been unmeasured differences in quality or environmental exposure between the chicks that caused the variation both in telomere dynamics and in position within the weight hierarchies of the larger broods.

Our results add data from a novel model system to an increasingly broad range of evidence that early-life adversity of a type that can be broadly characterized as ‘psychosocial’ – in this case, exerted on chicks by the presence of larger competitors in the nest – can exert profound effects on the individual's somatic state. Telomere length is emerging as a key marker of such effects. A range of findings in humans and as well as other animals all suggest that, as a recent review put it, “telomeres powerfully quantify life's insults” [58]. Here, experiencing just 12 days of competition with heavier nestmates in an animal that can live for many years was enough to cause a measurable difference in TL. Since TL has been associated with adult health and survival across a number of studies [11], [15]–[21], it is likely to become central to the quest to understand how early-life adversity gets under the skin, and how it can continue to influence health years after it occurs. Our finding that it is position within the size hierarchy, rather than absolute size per se, that is important is of particular interest since subordinate social positions have been associated with poor health outcomes in humans in a wide range of contexts [59], [60]. The extent to which those associations arise through similar pathways to those involved in starling chick development is of course not clear. However, it is possible that experimental paradigms such as the present one, as well as shedding light on how early life environment can determine future fitness prospects in wild birds, will also be useful as models for the effects of disadvantage on health in humans.

## Supporting Information

File S1 **Raw data from the study.**
(CSV)
